# Improving Eye Motion Sequence Recognition Using Electrooculography Based on Context-Dependent HMM

**DOI:** 10.1155/2016/6898031

**Published:** 2016-09-27

**Authors:** Fuming Fang, Takahiro Shinozaki, Yasuo Horiuchi, Shingo Kuroiwa, Sadaoki Furui, Toshimitsu Musha

**Affiliations:** ^1^Department of Information Processing, Tokyo Institute of Technology, Yokohama, Japan; ^2^Division of Information Sciences, Chiba University, Chiba, Japan; ^3^Department of Computer Science, Tokyo Institute of Technology, Tokyo, Japan; ^4^Brain Functions Laboratory Inc., Yokohama, Japan

## Abstract

Eye motion-based human-machine interfaces are used to provide a means of communication for those who can move nothing but their eyes because of injury or disease. To detect eye motions, electrooculography (EOG) is used. For efficient communication, the input speed is critical. However, it is difficult for conventional EOG recognition methods to accurately recognize fast, sequentially input eye motions because adjacent eye motions influence each other. In this paper, we propose a context-dependent hidden Markov model- (HMM-) based EOG modeling approach that uses separate models for identical eye motions with different contexts. Because the influence of adjacent eye motions is explicitly modeled, higher recognition accuracy is achieved. Additionally, we propose a method of user adaptation based on a user-independent EOG model to investigate the trade-off between recognition accuracy and the amount of user-dependent data required for HMM training. Experimental results show that when the proposed context-dependent HMMs are used, the character error rate (CER) is significantly reduced compared with the conventional baseline under user-dependent conditions, from 36.0 to 1.3%. Although the CER increases again to 17.3% when the context-dependent but user-independent HMMs are used, it can be reduced to 7.3% by applying the proposed user adaptation method.

## 1. Introduction

Eye motion-based human-machine interfaces are used to provide a means of communication for those who can move nothing but their eyes because of injury or disease, such as amyotrophic lateral sclerosis (ALS) [1, 2]. As an example of such a system, Ohya et al. have proposed an eye-blinking-based software keyboard system [3] in which a user can specify an arbitrary column on the keyboard by blinking when a horizontally moving cursor reaches the desired column. Then, the user can similarly specify an arbitrary row by blinking when a vertically moving cursor reaches the desired row. Takahashi et al. have reported a command recognition system for a hands-free manipulation system [4]. In their system, blinks and eye motions are mapped to commands (e.g., go forward, turn left, and turn right) to control devices such as powered wheelchairs and robots. Aziz et al. have also developed a similar eye motion-based wheelchair navigation system [5].

To detect eye motions, electrooculography (EOG) is used, which is a type of weak biomedical signal captured by electrodes attached to the skin around the eyes [6]. For the recognition of eye motions from these signals, several approaches have been investigated. To detect an isolated eye motion event such as blinking, a threshold is used to determine whether amplitude of the signal is larger than a predefined constant [3]. When an event or a command is represented by a sequence of eye motions, a mechanism to recognize such sequences is required. For this purpose, some systems [4] use dynamic programming- (DP-) matching [7], whereas others [5, 8, 9] use hidden Markov models (HMMs) [10]. Compared with DP-matching, HMMs use probability distributions and have the advantage that they can model the variance of the input signals for better recognition performance.

To provide an efficient communication method, we have previously proposed an eye-to-speech modality conversion system [11] using an HMM-based EOG decoder. In this system, a recognition module recognizes a sequence of eye motions and converts it into a sequence of phonetic letters or characters, which is then sent to a speech synthesizer module to synthesize speech utterances. We have demonstrated that this system functions successfully for several basic inputs. However, the system faces difficulty in accurately recognizing the input eye motion sequences. This is because users tend to continuously input multiple eye motions to input the desired pronunciation of arbitrary utterances at relatively high speed. Because adjacent motions become fused together and the boundaries between them become unclear, the EOG signal is distorted and recognition becomes very difficult.

In this work, we improve HMM-based eye motion recognition to achieve higher recognition accuracy for continuous eye motion input. The fundamental idea is to explicitly model the context information of eye motions using context-dependent eye motion HMMs; this approach is analogous to the triphone model used in high-performance speech recognition systems [12]. By using the context information, the changes in the EOG signals caused by the fusing of eye motions can be explicitly modeled, enabling more robust recognition with respect to this distortion. In general, the parameters of the HMMs must be estimated before they can be used for decoding. For higher recognition accuracy, a user-specific HMM-based EOG model should be trained using a set of EOG data collected from the same user. However, if a user-independent model could be used with only a small amount of user-specific EOG data, it would be more convenient for the user because it would reduce the effort required to record EOG data to generate a customized EOG model. To address this possibility, we propose a method for the user adaptation of a user-independent HMM-based EOG model and investigate the trade-off between recognition accuracy and the required amount of user-dependent EOG data.

The organization of this paper is as follows. In Section 2, the basics of EOG are briefly reviewed. In Section 3, an overview of the eye-to-speech system is provided.  Section 4 presents the proposed context-dependent modeling methods for EOG-based eye motion recognition and the method for the user adaptation of a user-independent HMM-based EOG model. Sections 5 and 6 introduce the EOG database and experimental setup, respectively.  Section 7 discusses the experimental results. Finally, Section 8 provides the conclusion and suggestions for future studies.

## 2. Electrooculography (EOG)

**Figure 1:**
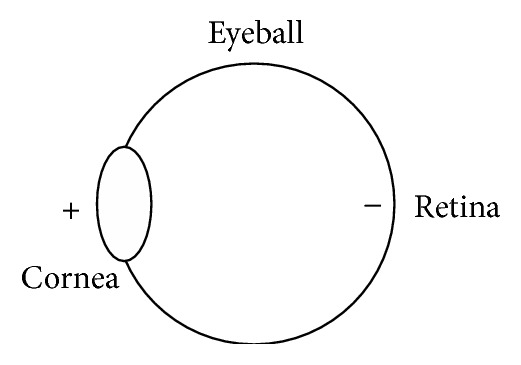
Corneoretinal potential.

An electrical potential exists in the eyeball between the cornea and retina, as shown in Figure 1. This potential is called the corneoretinal potential (CRP). The cornea side has a positive charge, and the retina side has a negative charge. The CRP can be observed via EOG by means of electrodes attached to the skin around the eyes. The EOG varies with different eye movements and can therefore be used to determine the position of the eye. The magnitude of the EOG is approximately 290 to 1,100 *μ*V/rad. The frequency is approximately 0 to 30 Hz [6, 9]. EOG-based eye motion detection functions even when the eyes are closed. It has been reported that EOG signals do not exhibit large differences between able-bodied persons and ALS patients [13]. EOG detectors are noninvasive and simpler than those for electroencephalography (EEG) [14] because EOG signals are larger in magnitude and easier to observe.

Potential noise sources for EOG include changing of the electrical contact conditions between electrodes and skin due to comovement of mimetic muscles and eyes, artifact caused by myoelectricity, and electromagnetic radiation from the power line. The first two noises are due to muscle activities and are expected to become smaller for ALS patients, and the third noise is independent of the users.

## 3. Eye-to-Speech System

**Figure 2:**
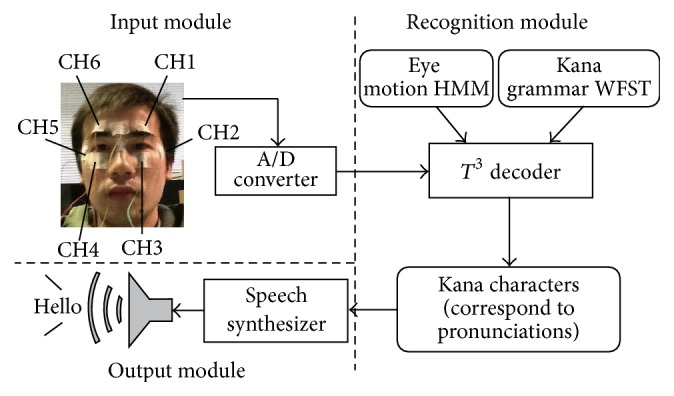
Overview of the eye-to-speech system.

Figure 2 shows an overview of our proposed eye-to-speech system [11]. This system consists of three modules: an input module, a recognition module, and an output module. The input module detects the EOG signal via electrodes attached to the skin around the eyes. The detected EOG signal is amplified and digitized and is then sent to the recognition module. The recognition module is an HMM-based decoder and converts the EOG signal into a sequence of characters in accordance with a predefined input protocol. Finally, the output module synthesizes a speech waveform from the identified characters and outputs it via a loudspeaker. The details of the system are as follows.

### 3.1. EOG Detection

**Figure 3:**
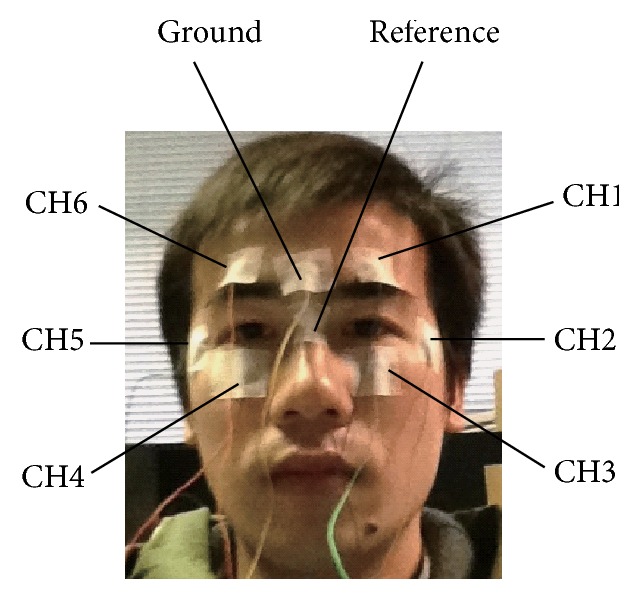
Location of the electrodes for EOG detection.

Eight electrodes are used to detect the EOG signal. Among them, one is a ground electrode, another is a reference electrode, and the others are measurement electrodes. The locations of these electrodes are shown in Figure 3. Regarding the measurement electrodes, two (CH1 and CH6) are attached above the eyes, another two (CH3 and CH4) are attached below the eyes, and the remaining two (CH2 and CH5) are attached at the left and right sides of the eyes. The ground electrode is attached to the forehead, and the reference electrode is attached between the eyes.

**Figure 4:**
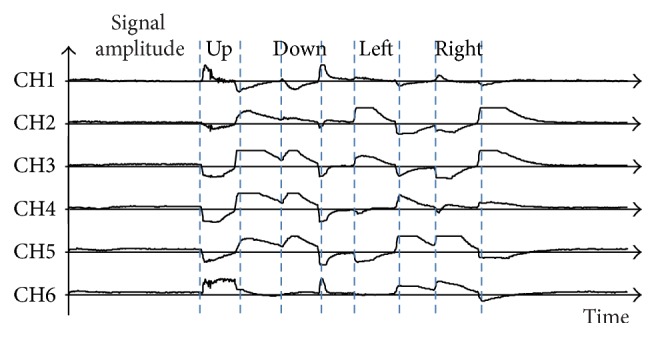
An example of an EOG signal. This signal was obtained using the eye-to-speech system and corresponds to a motion sequence consisting of up, center, down, center, left, center, right, and center motions.

In this study, five types of eye motions were used to input the desired characters: “up,” “down,” “left,” “right,” and “center.” A “center” motion is looking straight ahead. These motions are relative to the users' face and are not affected by the posture.  Figure 4 shows an example of an EOG signal obtained using the eye-to-speech system. This signal corresponds to a motion sequence consisting of up, center, down, center, left, center, right, and center. As can be observed in this example, the recorded signal reflects the eye movements that generated it.

### 3.2. EOG Recognition

In the eye-to-speech system, HMMs are used to construct EOG models, and the *T* ^3^ speech recognition decoder [15, 16] is employed for eye motion recognition. *T* ^3^ supports live decoding, and a partial output is obtained when a prefix of a recognition result is determined. This means that even when a long utterance is input, the recognition result is continuously output from the beginning without waiting for the end of the input to arrive. A six-dimensional feature vector is formed from the six channels of the EOG signal, and a sequence of such vectors is input to the decoder.

### 3.3. Input Protocol

The number of different characters depends on the language to be used, but it is typically larger than the number of different eye motions. When the number of characters is larger than the number of eye motions, multiple motions must be combined to express a single character. The purpose of the input protocol is to define a mapping from eye motions to characters.

The characters used in our system are Japanese Kana, a Japanese phonetic alphabet. The Kana system consists of 48 basic characters and two modification marks that are similar to the umlaut. A combination of four eye motions is used to express these basic characters and the modification marks. As an example, a part of the input protocol is given in Table 1, in which /ga/ is a derived character, the first four motions correspond to a modification mark, and the last four motions correspond to the basic character /ka/. Because of the difficulty of recognizing boundaries between sequences consisting of the same eye motion, consecutive motions such as “up” followed by “up” (in which the eyes would remain looking up) are avoided in the protocol.

## 4. Proposed Method

### 4.1. EOG Modeling Using Context-Dependent HMMs

**Figure 5:**
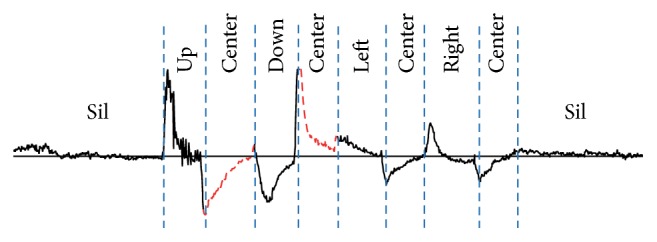
An example of a context-dependent EOG signal. Because the contexts of the first and second “center” motions differ, the shapes of their corresponding signals also differ. This sample signal was acquired from CH1. “sil” means that no motions were performed.

The simplest HMM-based approach for EOG modeling is to construct an HMM for each motion. However, we have found that the EOG signals for the same motion differ for different contexts. For example, a sample signal from CH1 is shown in Figure 5, in which the signal shapes corresponding to the first and second “center” motions are completely different. The gradient of the first shape is increasing, whereas the gradient of the second shape is decreasing. Therefore, if we were to model this eye motion with a single HMM, different characteristics would be mixed in the same model and accurate recognition would be difficult.

To address this problem, we propose a context-dependent eye motion HMM that is analogous to the context-dependent phone models used in high-performance speech recognition systems [12]. By considering the preceding eye motion context, multiple eye motion HMMs are prepared for the same eye motion depending on the context. We refer to such models as bi-eye motion HMMs because a different “p − t” HMM is prepared for each pair of eye motions (p, t), where “p” is the preceding context and “t” is the recognition target. Similarly, when both the preceding and succeeding eye motion contexts are considered, multiple eye motion HMMs are prepared for the same eye motion corresponding to the preceding and succeeding contexts. We refer to such models as tri-eye motion HMMs because a different “p − t + s” HMM is prepared for each triple of eye motions (p, t, s), where “p” is the preceding context, “s” is the succeeding context, and “t” is the recognition target. Because context-dependent HMMs explicitly model contextual effects, they are expected to enable more accurate recognition than context-independent HMMs. We refer to the original context-independent models as mono-eye motion HMMs.  Table 2 shows an example of the use of these context-independent and context-dependent models to represent a motion sequence.

**Figure 6:**
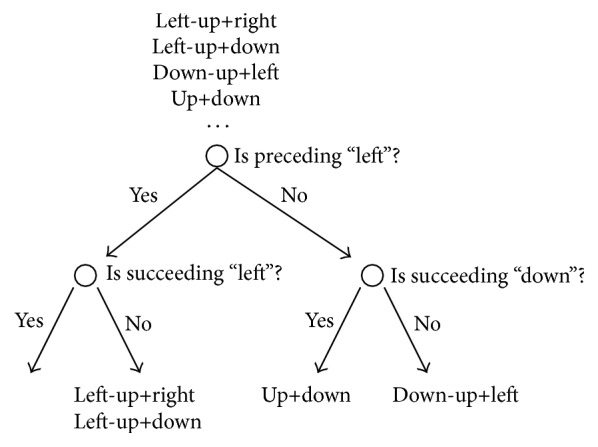
Decision tree-based state clustering for tri-eye motion unit parameter tying.

An HMM consists of mixtures of Gaussians representing emission probabilities and transition probabilities, and the parameters of these probability distributions must be estimated from training data. Because context-dependent models contain more parameters than context-independent models, they require more training data for accurate parameter estimation. In particular, tri-eye motion HMMs require a large amount of training data because the number of tri-eye motion HMMs is on the order of the number of eye motions cubed. To reduce the amount of training data required to obtain robust models, we cluster similar model parameters and then share data among them during the training process. To cluster the parameters, the decision-tree-based state tying approach [17] is used.  Figure 6 shows an example of the clustering process: a group of models for the same motion with different contexts is placed at the root of the decision tree, and a set of predefined questions are then answered to split these models until the change in likelihood before and after splitting is less than a predefined threshold. By applying decision-tree-based state tying, the balance between model complexity and estimation accuracy can be adjusted.

### 4.2. User Adaptation

**Figure 7:**
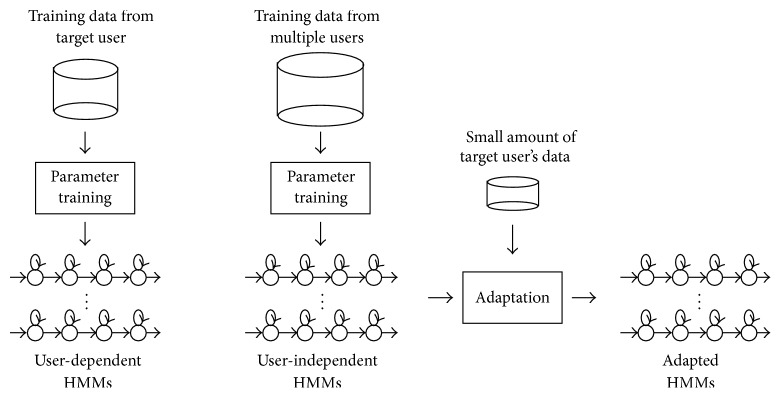
Training and adaptation processes for user-dependent, user-independent, and user adapted HMMs.

The parameters of the HMMs are estimated using a set of EOG training data. In general, when a statistical model is trained for a specific user using data from that user, it is called a user-dependent model. By contrast, when a model is trained on a mixture of data from multiple persons who are different from the target user, it is called a user-independent model.  Figure 7 outlines these two training processes. A user-dependent model typically provides higher recognition accuracy than a user-independent model because user-specific characteristics are precisely modeled. However, a user-independent model has the advantage that any user can use the model once it is trained. It might be possible to compensate for its low recognition performance to some degree by applying the speaker adaptation techniques used in speech recognition. To investigate this possibility, we propose the application of the maximum likelihood linear regression (MLLR) [18] and maximum a posteriori (MAP) [19, 20] adaptation techniques used in speech recognition. Both of these techniques are known to be very effective in improving speech recognition performance.

In addition to the HMM parameters, HMM-based continuous decoders usually possess a decoding parameter called an “insertion penalty,” which is used to control the balance between insertion and deletion errors. These errors are described in detail later in Section 6.1; in brief, they are related to the numbers of words that are mistakenly added to and deleted from the recognition results, respectively. We conjecture that optimizing the insertion penalty for each user should be a useful means of compensating for individual differences in input eye motion speed. Therefore, as a part of our proposed user adaptation method, we tune the insertion penalty for each user in addition to adapting the HMM parameters.

## 5. Database

The EOG data used in the experiments were recorded from five healthy participants. Four were male, one was female, and all were in their twenties. The recording system was operated as an independent system using batteries. Silver-silver chloride electrodes were used and fixed to the skin with conductive paste and tape. The participants were sitting on a chair during the recording. The sampling frequency for the A/D conversion of the EOG signals was 100 Hz. All data were recorded with the consent of the participants after they were informed about the recording process and the uses of the recorded data.

### 5.1. Training Data

The participants were asked to move their eyes in motion sequences in accordance with voice instructions to record the training data. One unit of training data was defined as a set of 22 motion sequences to comprise nearly all possible context combinations. The lengths of the sequences were between four and eight motions (e.g., “up, down, up, down, left, center”), and each unit contained 136 motions in total. A duration of approximately seven minutes on average was required to record one unit of training data, including the preparation of the electrodes and the rest period. To record 50 units per participant, the recording period was split into four or five separate sessions, with five to fifteen units recorded in each. All electrodes were attached and removed in every session. The total duration of the recorded data amounted to 860.7 minutes (14.3 hours).

### 5.2. Test Data

To record the test data, we selected 10 words representative of those commonly used by patients. Prior to recording, the participants were instructed to remember the corresponding motions for those words and then move their eyes in accordance with their memory to record the test data. Each participant recorded each word 10 times. If their eye motions were incorrect, they were asked to record again until all motions were correct.  Table 3 summarizes the details of the test data. Only one participant required three discontinuous sessions to record; the others completed their recording in one session. In total, the test data from all participants amounted to 118.1 minutes (2.0 hours) of data.

## 6. Experimental Setup

In this research, we focus on the performance of the EOG recognition. The mono-eye motion HMMs serve as the baseline, and the bi- and tri-eye motion HMMs are the proposed methods. The recognition experiments were performed offline. The real time factor (RTF) was less than 0.2 using an Intel Core i7 CPU.

### 6.1. Evaluation Measure

The recognition performance is measured in terms of the character error rate (CER), which is the ratio of incorrect characters in the recognition result to the total number of characters in the reference. For continuous character recognition, in which the total number of characters in an input sequence is unknown, three types of errors can be identified: substitution errors, deletion errors, and insertion errors. A substitution error means that the recognized character is different from the true character. A deletion error means that no character is output despite the existence of a character in the reference at the corresponding location. By contrast, an insertion error means that a character is inserted at a location where no character is present in the reference. Equation (1) gives the definition of the CER.

(1) $$\begin{array}{c} CER=\frac{S+D+I}{N}, \end{array}$$

where S, D, and I are the numbers of substitution, deletion, and insertion errors, respectively, and *N* is the total number of characters in the reference. For Kana recognition, the Kana CER is used.

### 6.2. HMM Training

The parameters of the HMMs were estimated using the training set described in Section 5. User-dependent models were used unless otherwise noted because higher recognition performance is expected from such models compared with user-independent models.

Four states were defined for each eye motion HMM, with a left-to-right topology. The emission distribution for each state was modeled as a mixture of Gaussians with 16 components. The observation features were 6-dimensional, and the elements were drawn from the corresponding EOG measurement channels. For the proposed tri-eye motion unit, the original models had 364 states (theoretically, there are (# states per HMM) × (# tri-eye motion models + # left bi-eye motion models + # right bi-eye motion models) = 4 × (4 × 5 × 4 + 4 × 5 + 5 × 4) = 480 possible states, but some of them did not appear in the training set); these were reduced to approximately 115 after decision-tree-based clustering. The threshold used as the termination criterion for the clustering process was determined based on a preliminary experiment. The hidden Markov model toolkit (HTK) [21] was used for model training and adaptation.

### 6.3. *N*-Gram Training

To achieve high recognition performance, *N*-gram language models [22] were used in combination with the EOG-based eye motion HMMs. The language models were character *N*-grams trained using texts from the Corpus of Spontaneous Japanese (CSJ) [23], which contains 12.4 million Kana characters. The vocabulary size was 70, which is equal to the total number of different Kana characters. The *N*-gram orders *N* were chosen to be 1, 2, and 3. The CMU-Cambridge SLM toolkit [24] was used to train the character *N*-grams.

The performance of language models is measured in terms of perplexity [25]. The value of this measure can be interpreted as an averaged divergence of possibility. A smaller perplexity value indicates better performance.  Table 4 shows the perplexity of the character *N*-grams.

## 7. Results

### 7.1. Preliminary Experiment

As a preliminary experiment, the recognition performances of the threshold-based and HMM-based approaches were compared. For threshold-based recognition, the difference between the left (CH2) and right (CH5) EOG signals was used to detect horizontal eye motions. For vertical eye motion detection, the difference between the average of CH1 and CH6 and the average of CH3 and CH4 was used because the CH1 and CH6 electrodes were attached above the eyes and the CH3 and CH4 electrodes were attached below the eyes. If the norm of the resulting two-dimensional EOG vector was smaller than a predefined threshold, then the signal was recognized as indicative of a “center” motion. Otherwise, the inner products of the EOG vector and unit vectors in four directions, (1,0), (0,1), (−1,0), and (0, −1), were computed, and the signal was recognized as indicative of the one of the four directions (“left,” “up,” “right,” or “down”) that yielded the largest inner product. These decisions were made at the same frequency as the sampling rate, which was 100 Hz in our experiment. However, the results were sometimes unstable and noisy. Therefore, a postfilter was applied in which voting was performed with a 1000 msec window width and a 10 msec window shift, and the most frequently identified motion was output. The final recognition result was obtained by merging consecutive motions into a single event. For HMM-based recognition, the same two-dimensional vector as in the threshold-based recognition was used in addition to the six-dimensional vector for comparison purposes. The HMMs used were the conventional context-independent models, and they were trained under user-dependent conditions.

Table 5 shows the recognition results. The performance was measured in terms of the error rate in motion recognition, which is a simpler task than character recognition because each character is represented by a combination of multiple motions. The definition of the motion error rate is similar to that of the CER; however, the units are not characters but rather the five types of eye motions. As shown in the table, the error rate of the threshold-based method was 46.5%, which is large. This is because the EOG signal contained electrical noise and artifacts such as muscle potentials and distortion due to continuous eye movement. When the HMM-based approach was used with the same two-dimensional input signal, the error rate was considerably reduced to 23.6%. This was because HMMs consider the distribution of the observations and are robust to noise. When the six-dimensional input was used, the error rate was further reduced to 11.9%, demonstrating the advantage of HMMs for robust recognition.

### 7.2. Recognition Using Context-Dependent HMMs and *N*-Grams

**Figure 8:**
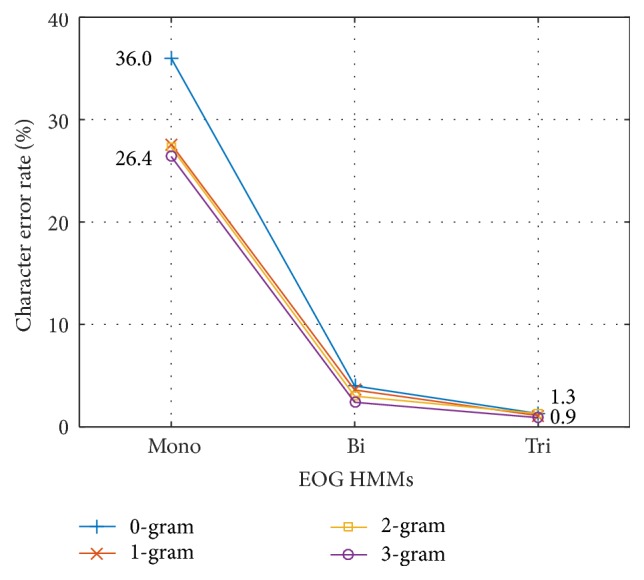
Kana CER of mono-, bi-, and tri-eye motion unit and different orders of *N*-grams.

Figure 8 shows the CERs obtained using the mono-, bi-, and tri-eye motion units and *N*-grams of different orders, where 0-gram means that no language model was used. The HMMs were user-dependent and were trained using all 50 units of training data recorded for each user. As is evident, the context-dependent EOG models yielded substantially better results compared with the context-independent model regardless of the *N*-gram order. When *N*-grams were not used (i.e., the 0-gram case), the proposed tri-eye motion unit achieved a significant reduction in relative error rate of 96.4% compared with the mono-eye motion unit, from a CER of 36.0% to a CER of 1.3%. This was because the EOG models were constructed based on different contexts and could clearly distinguish the different characteristics of the EOG signals. The error rate also decreased as the *N*-gram order increased regardless of the type of EOG model used. This finding suggests that the use of *N*-grams can also contribute to reducing the recognition error rate. The lowest error rate of 0.9% was achieved when the tri-eye motion unit was used in conjunction with the 3-gram.

### 7.3. User Adaptation

For the user adaptation experiments, user-independent tri-eye motion EOG HMMs were trained using the 200 units of training data from four of the five participants, excluding the target user for adaptation. To adapt the resultant user-independent HMM-based EOG model to the target user, the training data from the target user was used as the adaptation data for MLLR and MAP adaptation. To investigate the changes in the recognition error rate with different amounts of adaptation data, adaptations were performed using different randomly selected subsets of the training data, where the amounts of adaptation data selected were 0.2, 0.3, 0.5, 1.0, 2.0, and 5.0 units. To ensure reliable results, the experiments were repeated 10 times for each condition using independently sampled adaptation data. The experiments were also repeated with each of the five participants as the adaptation target. Therefore, 50 ( = 5 × 10) adaptations in total were performed for each condition, and the results were averaged.

**Figure 9:**
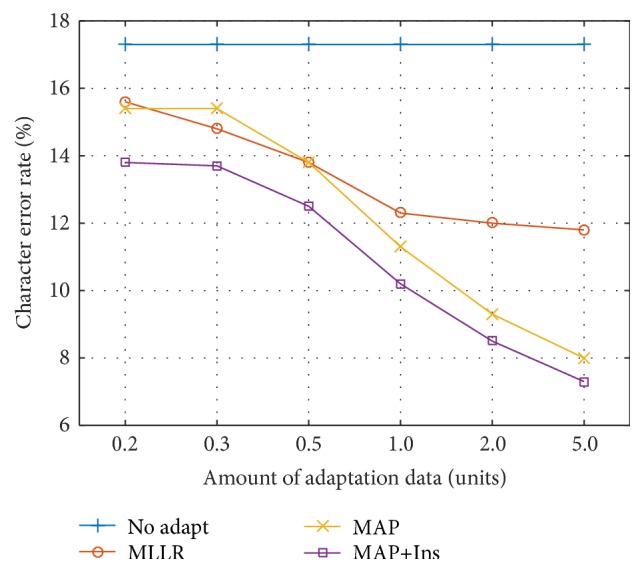
Kana CER with the user-independent system and adaptation.

Figure 9 shows the user adaptation results evaluated on the test data. For the language model, 3-gram model was used. In the figure, “No adapt” represents the results for the user-independent model without adaptation. Because of between-user differences in the EOG signals, such as different amplitudes and different speeds of eye motion, the CER for the user-independent model (17.3%) was substantially higher than those for the user-dependent models. When adaptation was performed using the MLLR or MAP technique, the error rate was considerably reduced. This is because the tendencies of the specific users were incorporated into the original user-independent model via this adaptation. It was also observed that the error rate decreased with an increasing amount of adaptation data used. The MAP adaptation was more advantageous than the MLLR adaptation, resulting in similar or superior recognition performance.

The results of the combination of MAP adaptation with insertion penalty tuning are also shown in the figure, labeled as “MAP+Ins.” As seen, further improvement was achieved compared with MAP adaptation alone. When five units of adaptation data were used, the CERs of the MLLR-, MAP-, and MAP+Ins-based adaptations were 11.8, 8.0, and 7.3%, respectively. These values correspond to relative reductions compared with the 17.3% CER of the user-independent model of 31.8, 53.8, and 57.8%, respectively.

## 8. Conclusion and Future Work

Eye motion-based human-machine interfaces based on EOG provide a vital communication channel for those who can move nothing but their eyes because of injury or disease, such as ALS. To establish a robust communication channel that can transmit not only simple signs but also arbitrary utterances, accurate EOG recognition for sequential eye motions is required. However, it is difficult to achieve good recognition accuracy using the conventional threshold-based method or HMM-based methods using context-independent models because the signal is considerably distorted by the context effects of adjacent eye motions.

In this paper, we proposed a context-dependent HMM modeling approach that uses separate HMMs for the same eye motion with different contexts. More specifically, we proposed bi-eye motion HMMs that are conditioned by the preceding eye motion context and tri-eye motion HMMs that are conditioned by both the preceding and succeeding eye motion contexts. The HMM parameters, that is, the parameters for mixtures of Gaussians representing emission distributions and transition probabilities, are estimated from training data. The number of parameters of context-dependent models is larger than that of context-independent models. In particular, the number of parameters of the tri-eye motion HMMs is proportional to the number of eye motions cubed, and accurate parameter estimation from a limited amount of training data becomes difficult. To address this problem, decision-tree-based state clustering is applied to balance the model complexity and the accuracy of parameter estimation. Because EOG signals vary from person to person, the HMM-based EOG models were trained under user-dependent conditions. However, it would be useful if a user-independent model could be adopted and trained with only a small amount of user-specific data. To investigate this possibility, various approaches to user adaptation were also evaluated.

The experimental results showed that the recognition performance measured in terms of the Kana CER was considerably reduced compared with the conventional context-independent mono-eye motion HMM approach, from 36.0 to 1.3%, when the proposed context-dependent tri-eye motion HMM-based approach was used. This result corresponds to a relative CER reduction of 96.4%. When this approach was combined with a 3-gram-based language model, the CER was further reduced to 0.9%, and highly accurate EOG recognition for sequential eye motions was achieved. In the user adaptation experiment, it was shown that a combination of MAP adaptation with insertion penalty tuning is the most effective approach. With this adaptation method, a relative CER reduction of 57.8%, from 17.3 to 7.3%, was achieved compared with the user-independent model.

One of the possible difficulties when our approach is applied to real end-users is that many combinations to be able to produce different characters may be difficult to learn in a short time. To solve this problem, we have developed a protocol recommendation method that produces an easily learnable protocol customized for each user based on a reduced character table [26]. Another difficulty may be that if the eye motions are affected by the stages of progression of the illness, the EOG signal deviates from the distribution modeled by the HMM, and it causes degradation in the recognition performance. For this problem, application of user adaptation is expected to be useful in updating the HMM according to the progression of the illness.

Future work includes fabrication of a portable hardware system implementing the proposed eye-to-speech communication technique and trials with ALS patients to validate the technology and study usability issues among this population.

## Figures and Tables

**Table 1:** Part of the input protocol for the eye-to-speech system.

Kana character in alphabet	Corresponding eye motions
/a/	Up, down, up, center
/ko/	Up, center, up, center
/e/	Up, left, up, center
/ka/	Up, left, right, center
/ga/	Right, left, down, center, up, left, right, center

**Table 2:** An example of the use of mono-, bi-, and tri-eye motion units to represent the motion sequence “up, down, up, center.” “p − t” means that “p” is the motion preceding the target motion “t” and “t + s” means that “s” is the motion succeeding “t.”

Type of unit	Corresponding representation
Mono	Up, down, up, center
Bi	Up, up-down, down-up, up-center
Tri	Up + down, up-down + up, down-up + center, up-center

**Table 3:** Details of the test data recorded for each participant.

Kana word (meaning in English)	Length (# of motions)	Recorded data (# of units)
Yes	8	10
No	12	10
Thanks	24	10
Hello	20	10
Sore	12	10
Stomach	12	10
Head	12	10
Foot	8	10
Water	12	10
Television	16	10

Total	136	100

**Table 4:** Perplexity of character *N*-grams.

*N*-grams	1-gram	2-gram	3-gram

Perplexity	21.49	21.39	17.19

**Table 5:** Eye motion recognition error rates for the threshold- and HMM-based methods.

Recognition method	Error rate (%)
Threshold	46.5
HMM (2 channels)	23.6
HMM (6 channels)	11.9
